# Impact of antipsychotics, antiparkinsonian drugs, and anthraquinone stimulant laxatives on severe constipation in patients with schizophrenia

**DOI:** 10.1002/pcn5.70255

**Published:** 2025-11-21

**Authors:** Toshiaki Nadaya, Tokuya Inaguma, Tomoko Nagao, Takuto Ishida

**Affiliations:** ^1^ Department of Pharmacy Tokyo Metropolitan Matsuzawa Hospital Tokyo Japan; ^2^ Department of Psychiatry Tokyo Metropolitan Matsuzawa Hospital Tokyo Japan; ^3^ Department of Internal Medicine Tokyo Metropolitan Matsuzawa Hospital Tokyo Japan

**Keywords:** antipsychotics, constipation, laxatives, schizophrenia

## Abstract

**Aim:**

Constipation in schizophrenia patients receiving antipsychotics can lead to severe intestinal complications, such as paralytic ileus or megacolon. Despite the clinical importance of the topic, few studies have thoroughly investigated the risk factors of constipation‐related complications. Therefore, the present study aimed to identify clinical and pharmacological factors associated with severe, constipation‐related conditions in patients with schizophrenia.

**Methods:**

The present, retrospective study analyzed inpatients with schizophrenia and constipation at Tokyo Metropolitan Matsuzawa Hospital between 2014 and 2018. Logistic regression analysis was performed to test for any association between severe, constipation‐related conditions and patient demographics and the use of various antipsychotics, antiparkinsonian drugs, and anthraquinone stimulant laxatives.

**Results:**

Of the 4114 patients included, 556 had a severe, constipation‐related condition. High dosages of clozapine (odds ratio [OR]: 8.688; 95% confidence interval [CI]: 1.926–39.186; *p* = 0.005), haloperidol (OR: 1.987; 95% CI: 1.093–3.610; *p* = 0.024), anthraquinone stimulant laxatives (OR: 1.769; 95% CI: 1.337–2.340; *p* < 0.001), antiparkinsonian drugs (OR: 1.641; 95% CI: 1.280–2.104; *p* < 0.001), and female sex (OR: 1.318; 95% CI: 1.097–1.582; *p* = 0.003) were found to be significantly associated with this condition. On the other hand, high‐dosage risperidone was associated with a reduced risk (OR: 0.688; 95% CI: 0.508–0.932; *p* = 0.016).

**Conclusion:**

These findings highlighted the need for careful monitoring of constipation and for optimizing antipsychotic drug regimens. The use of antiparkinsonian drugs and anthraquinone stimulant laxatives should be limited to the short term.

## Graphical abstract

This study investigated risk factors for severe constipation in patients with schizophrenia receiving pharmacological treatment. A retrospective analysis of over 4000 inpatients revealed that high‐dose use of clozapine, haloperidol, antiparkinsonian drugs, and anthraquinone stimulant laxatives significantly increased the risk. The findings highlight the importance of careful medication management to prevent serious gastrointestinal complications.

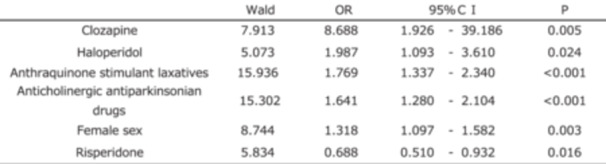

## INTRODUCTION

Constipation caused by antipsychotic agents can lead to severe intestinal complications, such as paralytic ileus or megacolon, making it a critical issue in psychiatric practice.^1^ While many studies have examined the relationship between antipsychotic agents and constipation, few have specifically explored the risk factors of severe constipation.^2^, ^3^, ^4^, ^5^, ^6^ Furthermore, there is a paucity of research on the relationship between constipation and the dosage of antipsychotic agents.^7^, ^8^, ^9^, ^10^ Many previous studies of severe constipation lacked comprehensiveness; for example, some only focused on specific drugs, such as clozapine or olanzapine,^7^, ^8^ while others focused only on cases of severe constipation that progressed to ileus.^9^, ^10^, ^11^

A retrospective cohort study by Chen et al., one of the most comprehensive studies of the risk factors of severe constipation,^12^ analyzed multiple antipsychotic agents using the Defined Daily Dose (DDD) as a dosage index and included ischemic bowel disease in the definition of severe constipation. However, the study classified the concomitant administration of anticholinergic agents as a confounder rather than as a risk factor and did not consider the impact of laxatives, which was a major limitation. Other studies of the relationship between antipsychotic agents and constipation have highlighted the anticholinergic effects of the drugs, which suppress bowel motility.^3^, ^4^ However, given the diverse receptor profile of antipsychotic agents, the risk of severe constipation may vary by drug and dosage.^11^, ^12^

Antiparkinsonian drugs, which are frequently co‐prescribed with antipsychotics, also possess anticholinergic properties that can contribute to chronic constipation. Additionally, long‐term administration of high‐dose anthraquinone stimulant laxatives is also known to cause chronic constipation.^13^ Since both the effectiveness and side effects of medications follow a dose–response relationship, it is essential to consider not only the type but also the dosage of drugs, especially in high‐dose regimens, when evaluating the constipation risk. The present study used logistic regression to test for any association between high‐dose regimens of specific drugs and the severity of constipation.

## METHODS

### Participants

The present, single‐center, retrospective, cohort study included inpatients with schizophrenia and the comorbidity of constipation at Tokyo Metropolitan Matsuzawa Hospital between 2014 and 2018. The patients with a diagnosis of both schizophrenia and constipation were extracted from the electronic medical records. Schizophrenia was diagnosed by psychiatrists using the ICD‐10 criteria. Severe constipation was defined as the occurrence of any of the following conditions selected by gastroenterologists from diagnostic entries in the electronic medical records: severe constipation, severe atonic constipation, paralytic constipation, intestinal paralysis, ileus, intestinal pseudo‐obstruction, megacolon, and ischemic colitis. Conditions that were clearly unrelated to drug use, such as mechanical ileus or strangulated ileus, were excluded.

### Study variables

The primary outcome was the occurrence of a severe, constipation‐related condition. Independent variables included age, sex, and medications, with the focus being placed on antipsychotics, antiparkinsonian drugs, and anthraquinone stimulant laxatives. In the severe constipation group, data on medication were obtained from prescriptions issued immediately before the diagnosis of any severe, constipation‐related condition. In the control group, data were collected from the period when the chlorpromazine equivalent dose attained its maximum value. Chlorpromazine equivalent dosages were calculated using the equivalence table of Inagaki et al.^14^, ^15^ regardless of the drug formulation (e.g., oral, transdermal patch, long‐acting injection). The dosage of antiparkinsonian drugs was converted to its biperiden equivalent,^14^ while that of anthraquinone stimulant laxatives was converted to its sennoside equivalent based on Okazaki's study.^16^ The Japanese Pharmacopoeia standard of 2.5 mg sennoside per gram of powdered medicinal rhubarb was applied for rhubarb‐containing, traditional medicines not covered by Okazaki (Table 1).

**Table 1:** Dosage equivalency conversion for anthraquinone stimulant laxatives.

Generic name	Brand name (formulations included in the study)	Sennoside equivalent dosage	Basis for conversion
Sennoside preparations	Pursennid® and generics	12 mg/tablet	Each tablet contains sennoside 12 mg
Senna seed and leaf products	Aloesen®	7.5 mg /0.5 g sachet	The average value based on the information in the package insert stating “contains 10–20 mg of sennoside A and B per gram” was used for conversion
Casanthranol and docusate combination products	Bimas® and Bencol®	1.32 mg/tablet	Based on Okazaki's evaluation,^16^ which estimated the stimulant component potency of one tablet to be 11% of one tablet of Pursennid®
Powdered medicinal rhubarb	Prescription kampo extracts	2.5 mg/g	Applied to kampo formulations containing rhubarb, defined by the Japanese Pharmacopoeia as “containing not less than 0.25% of sennoside A in the dried crude drug”

### Statistical analysis

Categorical variables were presented as a frequency and a percentage, while continuous variables were expressed as the median and an interquartile range. Comparison of variables between the patients and control subjects was performed using Fisher's exact test for categorical variables and the Mann–Whitney *U* test for continuous variables. The severe constipation group and control group were compared using the method below. Briefly, multiple logistic regression analysis was conducted to examine the effects of dichotomized drug dosages, age, and sex on the occurrence of severe, constipation‐related conditions.

The present study focused on the association between high‐dose regimens of individual drugs and the occurrence of severe, constipation‐related conditions. Therefore, the dosage of each antipsychotic, the total antiparkinsonian drug dosage, and the total anthraquinone stimulant laxative dosage were dichotomized using predefined threshold values, which were determined on the basis of generally accepted, safe dosage ranges. For antipsychotic agents, the threshold was 600 mg/day of the chlorpromazine equivalent corresponding to the dosage used to assess a poor drug response in treatment‐resistant schizophrenia.^17^ For antiparkinsonian drugs, the threshold was 2 mg/day of the biperiden equivalent, which aligned with the recommended starting dosage for oral biperiden. For anthraquinone stimulant laxatives, the threshold was 12 mg/day of the sennoside equivalent, the standard dosage for a single tablet of sennoside.

All statistical analyses were performed using spss version 26.0 (spss Inc., Chicago, IL, USA). All tests were two‐tailed, and *p* <0.05 was considered to indicate statistical significance. The study was reviewed and endorsed by the ethics committee of Tokyo Metropolitan Matsuzawa Hospital (2023‐02) and safeguarded the patients' privacy.

## RESULTS

In total, 4114 patients with schizophrenia were included. Of these, 556 had a severe, constipation‐related condition. Table 2 compares background data between the severe constipation group and the control group. The proportion of female patients was significantly higher in the severe constipation group than in the control group. No significant difference was observed in the median age between the groups. The proportion of high‐dose regimens of clozapine, haloperidol, antiparkinsonian drugs, and anthraquinone stimulant laxatives was significantly higher in the severe constipation group.

**Table 2:** Comparison of the severe constipation group and the control group by high‐dose regimen ratios.

	Severe constipation group (*n* = 556)	Control group (*n* = 3558)	*p*	
Male/female, *n*	247/309	1839/1719	0.013	
Age, years	52.5 (40.4–65.8)	52.2 (36.5–68.8)	0.998	
Antipsychotics				
Aripiprazole	8/76, 10.5%	78/702, 11.1%	0.248	
Asenapine	0/0, 0.0%	6/10, 60.0%	0.333	
Blonanserin	0/35, 0.0%	0/196, 0.0%	‐	
Brexpiprazole	0/1, 0.0%	0/17, 0.0%	‐	
Bromperidol	0/1, 0.0%	6/16, 37.5%	0.333	
Chlorpromazine	1/28, 3.6%	1/102, 1.0%	0.131	
Clozapine	4/7, 57.1%	3/5, 60.0%	<0.001	
Fluphenazine	0/4, 0.0%	1/12, 8.3%	0.693	
Haloperidol	16/52, 30.8%	45/185, 24.3%	0.003	
Levomepromazine	0/59, 0.0%	0/296, 0.0%	‐	
Nemonapride	0/0, 0.0%	2/5, 40.0%	0.576	
Olanzapine	68/151, 45.0%	545/963, 56.6%	0.057	
Paliperidone	12/24, 50.0%	80/195, 41.0%	0.894	
Perospirone	0/30, 0.0%	0/153, 0.0%	‐	
Perphenazine	8/8, 100.0%	0/7, 0.0%	‐	
Propericiazine	0/11, 0.0%	2/31, 6.5%	0.576	
Quetiapine	36/96, 37.5%	248/709, 35.0%	0.668	
Risperidone	59/210, 28.1%	461/1,443, 31.9%	0.122	
Sulpiride	0/14, 0.0%	0/37, 0.0%	‐	
Zotepine	2/70, 2.9%	17/353, 4.8%	0.703	
Anticholinergic antiparkinsonian drugs	105/147, 71.4%	453/733, 61.8%	<0.001	
Anthraquinone stimulant laxatives	75/129, 58.1%	266/457, 58.2%	<0.001	

*Note*: Gender is presented as male/female ratio, and age as median and quartile range. Drug data are presented as number of high‐dose cases/all cases and percentage within that group. Sample pools < 5 were omitted. Prescribed medications: antipsychotics, antiparkinsonian drugs, and anthraquinone stimulant laxatives.

Table 3 presents the results of logistic regression analysis of the factors associated with severe, constipation‐related conditions. Clozapine was most strongly associated with these conditions (odds ratio [OR]: 8.688; 95% confidence interval [CI]: 1.926–39.186; *p* = 0.005), followed by haloperidol (OR: 1.987; 95% CI: 1.093–3.610; *p* = 0.024), anthraquinone stimulant laxatives (OR: 1.769; 95% CI: 1.337–2.340; *p* < 0.001), antiparkinsonian drugs (OR: 1.641; 95% CI: 1.280–2.104; *p* < 0.001), and female sex (OR: 1.318; 95% CI: 1.097–1.582; *p* = 0.003). Risperidone, on the other hand, was associated with a reduced risk of severe constipation (OR: 0.688; 95% CI: 0.508–0.932; *p* = 0.016).

**Table 3:** Risk factors of severe constipation identified by logistic regression.

	Wald	OR	95% CI	*p*
Clozapine	7.913	8.688	1.926–39.186	0.005
Haloperidol	5.073	1.987	1.093–3.610	0.024
Anthraquinone stimulant laxatives	15.936	1.769	1.337–2.340	<0.001
Anticholinergic antiparkinsonian drugs	15.302	1.641	1.280–2.104	<0.001
Female sex	8.744	1.318	1.097–1.582	0.003
Risperidone	5.834	0.688	0.510–0.932	0.016

*Note*: Drug dosages were categorized into two groups based on predefined threshold values and analyzed using the likelihood ratio test by forward selection. An equivalent dosage was used in each category (chlorpromazine equivalent dosage for antipsychotics, the biperiden equivalent dosage for anticholinergic antiparkinsonian drugs, and the sennoside equivalent dosage for anthraquinone stimulant laxatives).

Abbreviations: CI, confidence interval; OR, odds ratio.

## DISCUSSION

The present study found that high‐dose clozapine, haloperidol, antiparkinsonian drugs, and anthraquinone stimulant laxatives as well as female sex were associated with the occurrence of severe, constipation‐related complications.

Many antipsychotic drugs, as well as antiparkinsonian drugs that are generally prescribed for drug‐induced Parkinsonism, exhibit anticholinergic effects. By blocking muscarinic receptors in the gastrointestinal tract, they inhibit intestinal peristalsis and defecation, physiological processes that are normally facilitated by parasympathetic dominance. In the present study, clozapine was strongly associated with severe constipation when administered at dosages exceeding 600 mg/day of the chlorpromazine equivalent (Table 3). Clozapine‐induced gastrointestinal hypomotility (CIGH) is a serious adverse effect characterized by extreme severity and a high mortality rate.^18^, ^19^ Although clozapine‐induced parasympathetic blockade strongly suppresses intestinal peristalsis, it should be noted that the worsening of CIGH is further influenced by multiple factors, including ethnicity, smoking status, sex, obesity, and concomitant medications. Asian patients require a lower dosage because of their lower metabolism, which is due to lower CYP1A2 activity. Previous studies of the blood concentration of clozapine recommended dosage adjustments in the range of 175–300 mg/day for Asian patients.^19^ In the present study, 58.3% (7/12) of the patients were receiving more than 300 mg/day of clozapine (equivalent to chlorpromazine 600 mg/day), and 57.1% (4/7) of these patients had severe constipation.

Previous studies found the CIGH incidence rate not only to be comparable to that of clozapine‐induced agranulocytosis but also to be associated with a higher mortality rate.^18^, ^20^ Therefore, blood concentration monitoring and bowel movement management are crucial to modulating clozapine administration.

Haloperidol was independently associated with severe constipation at a dosage exceeding 600 mg/day of the chlorpromazine equivalent (Table 3). Previous studies have reported a link between haloperidol administration and ileus development.^11^ It has also been suggested that high‐potency, first‐generation antipsychotic agents are associated with a higher incidence of ileus,^9^, ^12^ and one study found an increase in the hazard ratio after controlling for the effects of antiparkinsonian drugs with which they were commonly co‐administered.^12^ An inverse correlation has been found between the duration of physical activity under brightly lighted conditions and colonic transit time.^21^ Extrapyramidal symptoms caused by high‐potency, first‐generation antipsychotic agents may impair diurnal activity, potentially leading to reduced bowel motility. It should also be noted that haloperidol is not entirely free of anticholinergic effects, and blood concentration monitoring is useful for avoiding unintentional overdoses.^22^ Whenever the blood concentration of haloperidol is elevated, clinicians should consider the possibility that dopamine D2 receptor upregulation may have narrowed the optimal therapeutic window of the antipsychotic agents.^23^

Risperidone was independently associated with a lower risk of severe constipation at a dosage exceeding 600 mg/day of the chlorpromazine equivalent (Table 3). A study investigating the lifestyle and medication of patients with schizophrenia reported a significantly lower risk of constipation among those receiving risperidone.^5^ When prescribing an antipsychotic agent at a dosage exceeding 600 mg/day of the chlorpromazine equivalent, a drug having minimal anticholinergic activity should be chosen to prevent exacerbation of constipation. In addition to risperidone, paliperidone and aripiprazole also have a negligible anticholinergic effect.^22^ However, risperidone is more frequently used in clinical practice, thereby providing a larger sample pool and thus greater statistical power for analysis.

Antiparkinsonian drugs were independently associated with severe constipation when administered at a dosage exceeding 2 mg/day of the biperiden equivalent (Table 3). In the psychiatric setting, an increased anticholinergic burden has been linked to a higher risk of constipation.^3^, ^4^ Given that antiparkinsonian drugs with anticholinergic effects are well‐known contributors to constipation, our results suggested that these agents may play a role not only in the onset but also in the severe constipation‐related conditions.

Anthraquinone stimulant laxatives were independently associated with severe constipation at a dosage exceeding 12 mg/day of the sennoside equivalent (Table 3). As has recently been reported for magnesium oxide, laxatives may alter the gut microbiota and the production of short‐chain fatty acids.^24^ Animal studies of anthraquinone stimulant laxatives have also reported a reduction in beneficial bacteria, decreases in short‐chain fatty acids, and activation of inflammatory pathways.^25^ These findings may partly explain the association with severe constipation observed in our study. Numerous other studies claimed to find a relationship between these laxatives and the exacerbation of constipation; some have argued that the studies lacked robust evidence and that concerns regarding the harmful effects of these agents may have been overstated.^26^, ^27^ A conclusion to this debate has yet to be reached. However, the possibility that these agents may worsen constipation cannot be ruled out. Among the stimulant laxatives, sodium picosulfate solution is considered safer than anthraquinone stimulants for as‐needed use by the current guidelines.^28^, ^29^

Female sex was independently associated with severe constipation (Table 3). Chronic constipation is known to be more prevalent in women than in men,^26^, ^30^ and colonic functional decline due to aging tends to occur earlier in women.^31^ Idiopathic, chronic constipation also tends to be more severe in women,^32^ and female sex has been associated with an increased risk of ileus.^9^ In addition to differences in sex hormones and neural circuits, recent studies have reported sex‐specific differences in gut microbiota composition and metabolism.^33^, ^34^ These factors may collectively contribute to the higher risk of constipation observed in women. Furthermore, since the adoption of Rome IV, chronic constipation has come to be recognized as a facet of Disorders of Gut–Brain Interaction (DGBI), a multifactorial disease group involving gut–brain interactions, microbiota, hormones, and immune factors.^35^ The sex differences observed in the present study may also be understood within this framework.

## LIMITATIONS

The present study has several limitations. As an observational study, it provided a lower quality of evidence than interventional research. Additionally, unmeasured variables, such as body size and physical activity, may have acted as confounders. In this analysis, a uniform threshold of 600 mg/day of the chlorpromazine equivalent was used for all the antipsychotic agents. However, redefining the appropriate threshold for each drug might have affected the odds ratios. Furthermore, if the study period had been closer to the year 2025, recent changes in prescription trends, such as the increased use of clozapine and the introduction of newer antipsychotics, like asenapine and brexpiprazole, might have been taken on board and led to different findings. Similarly, the declining trend in the use of antiparkinsonian drugs and anthraquinone stimulant laxatives may also have affected the results. It should also be noted that patients receiving anthraquinone stimulant laxatives had already developed constipation and were therefore in a high‐risk state at the time of the study; thus, a causal relationship between anthraquinone stimulant laxatives and severe constipation cannot be conclusively determined. To understand better the relationship between anthraquinone stimulant laxatives and the risk of severe constipation, conversion values for non‐anthraquinone laxatives need to be established as a first step in conducting a comparative analysis.

## CONCLUSION

Among patients with schizophrenia, particular attention should be paid to the risk of severe constipation in those receiving high doses of agents such as clozapine, haloperidol, antiparkinsonian drugs, or anthraquinone stimulant laxatives, especially in women. Careful monitoring for constipation in daily clinical practice, rational selection and dose adjustment of antipsychotic agents based on appropriate prescribing strategies, and changes in the medication regimen when necessary are particularly important for these individuals. In addition, the use of antiparkinsonian drugs and anthraquinone stimulant laxatives should be limited to the short term.

## AUTHOR CONTRIBUTIONS

Toshiaki Nadaya and Tokuya Inaguma conceived the study design, conducted the analysis, and drafted the manuscript. Tomoko Nagao and Takuto Ishida contributed to data interpretation and critical revision of the manuscript. All authors approved the final version of the manuscript.

## CONFLICT OF INTEREST STATEMENT

The authors declare no conflicts of interest.

## ETHICS APPROVAL STATEMENT

The study was reviewed and endorsed by the Ethics Committee of Tokyo Metropolitan Matsuzawa Hospital (2023‐02) and conducted in accordance with the Declaration of Helsinki.

## PATIENT CONSENT STATEMENT

Patient consent was waived due to the retrospective nature of the study and the use of de‐identified data. The study protocol was approved by the institutional ethics committee.

## CLINICAL TRIAL REGISTRATION

Not applicable.

## Data Availability

The data that support the findings of this study are available on request from the corresponding author. The data are not publicly available due to privacy or ethical restrictions.
